# Instance Segmentation Based on Improved Self-Adaptive Normalization

**DOI:** 10.3390/s22124396

**Published:** 2022-06-10

**Authors:** Sen Yang, Xiaobao Wang, Qijuan Yang, Enzeng Dong, Shengzhi Du

**Affiliations:** 1 Tianjin Key Laboratory for Control Theory & Applications Complicated Systems, Tianjin University of Technology, Tianjin 300384, China; s_yang@email.tjut.edu.cn (S.Y.); wangxiaobao@stud.tjut.edu.cn (X.W.); qijuanyang2022@163.com (Q.Y.); dongenzeng@163.com (E.D.); 2 China Mobile Communications Group Jiangsu Co., Ltd., Suqian Branch, Suqian 223800, China; 3 Department of Mechanical Engineering, Tshwane University of Technology, Pretoria 0001, South Africa

**Keywords:** instance segmentation, normalized, batch size, self-adaptive normalization, adaptive weight

## Abstract

The single batch normalization (BN) method is commonly used in the instance segmentation algorithms. The batch size is concerned with some drawbacks. A too small sample batch size leads to a sharp drop in accuracy, but a too large batch may result in the memory overflow of graphic processing units (GPU). These problems make BN not feasible to some instance segmentation tasks with inappropriate batch sizes. The self-adaptive normalization (SN) method, with an adaptive weight loss layer, shows good performance in instance segmentation algorithms, such as the YOLACT. However, the parameter averaging mechanism in the SN method is prone to problems in the weight learning and assignment process. In response to such a problem, the paper proposes to replace the single BN with an adaptive weight loss layer in SN models, based on which a weight learning method is developed. The proposed method increases the input feature expression ability of the subsequent layers. By building a Pytorch deep learning framework, the proposed method is validated in the MS-COCO data set and Autonomous Driving Cityscapes data set. The experimental results prove that the proposed method is effective in processing samples independent from the batch size. The stable accuracy for all kinds of target segmentation is achieved, and the overall loss value is significantly reduced at the same time. The convergence speed of the network is also improved.

## 1. Introduction

The instance segmentation based on a deep convolutional neural network (CNN) is one of the important tasks of machine vision [1]. Results of instance segmentation contain semantic segmentation features for the classification at the pixel level and some of the characteristics for target detection. Deep learning often relies on a large number of data samples. However, the increase of samples reduces the convergence speed of the model and put forward higher requirements for GPU memory. Regarding the above-mentioned issues, normalizing the segmentation model, thereby restricting data distribution, will accelerate the training process and improve the accuracy.

Google developed a batch normalization (BN) method in 2015 [2], where the input features are aligned with a standard normal distribution. This method does not require the Dropout operation, and the statistics of the input features and output features can be kept the same, which speed up the iterative convergence speed of the network model in the training phase [2]. However, for the image instance segmentation, BN usually has to compromise between network model structure and sample batch size, which is not suitable for processing network models with large sample batch sizes. In 2016, the instance normalization (IN) was applied to image style transfer. The IN has good robustness to the low-level texture image features but is sensitive to high-level semantic features [3]. In the same year, Hinton et al. proposed a layer normalization method (LN), to make the data distribution of each layer in the network remain relatively stable in the training, meanwhile speeding up the training of the network model [4]. In 2018, a self-adaptive normalization (SN) was proposed by integrating BN, IN, and LN through learnable weights [5], which can deal with different segmentation tasks if proper weights are assigned to the three normalization methods. The SN was proved to be more robust to feature variety. However, the Softmax function in the SN has poor sparsity, making the learned weights easy to average. In fact, the three normalized weights are not very different; therefore, the segmentation accuracy of the SN is limited.

Given the drawbacks of the existing methods, it is necessary to enable the SN algorithm in more scenarios. Taking advantage of the SN and abandoning its shortcomings, this paper proposes an instance segmentation method based on an improved self-adaptive normalization. This method aims to achieve more reasonable and accurate weights by the learning mechanism in an adaptive weight loss layer. It is commonly recognized that the importance of a normalization method is determined by the generalization degree of the network model on the sample validation set. The proposed method effectively avoids the occurrence of averaging the weights of normalization methods, to ensure that the feature information processed by the main normalization method is not affected by the secondary normalization. Therefore, the feature expression ability of the subsequent network layer is improved.

In summary, our contributions are as follows:

1. We propose an adaptive normalization method that can autonomously assign weights by learning, and verify the effectiveness of our method on YOLACT++;
2. Our proposed adaptive normalization method can overcome the batch normalization problem under mini-batch sensitive conditions.

Based on the adaptive weight loss layer, a weight learning method is proposed to solve the problem of easy equalization of weight distribution in SN. Three experiments of 400 × 400, 550 × 550, and 700 × 700 on ResNet50, ResNet101, and DarkNet53 are conducted. The segmentation accuracy of the targets is improved, and the specific results are in Section 4.

In addition to the Introduction, this article consists of four other parts. In Section 2, several normalization methods and the adaptive normalization methods we propose are introduced, Section 3 discusses the methods of adaptive normalization layer weight learning, Section 4 gives the experiment and results, and Section 5 is the summary.

## 2. Related Work

### 2.1. Normalization Method

The training process of the neural network is mainly to learn the distribution law of data. When the distribution of each training sample batches is different, the iterative process of the entire network adapts to different distributions, which greatly reduces the training speed of the network. Normalization can be performed before the training samples are passed to the next layer of the neural network. By zooming and panning the samples, the mean and variance are assigned within a reasonable range [6,7]. By introducing a normalization layer, the sample data located in the saturated area of the loss function move to the unsaturated area. This is also equivalent to introducing noise in the neural network, enhancing the details of the output image, therefore improving the robustness of the network [8,9].

The general expression for normalization to obtain the mean and variance is shown in Equation (1):

(1) $$\mu =\frac{1}{\left|I\right|}\sum_{n,c,h,w}x_{nchw},\;\sigma^{2}=\frac{1}{\left|I\right|}\sum_{n,c,h,w}{\left(x_{nchw}-\mu \right)}^{2}$$

where n, c,h,w correspond to the integer coordinates in the four-dimensional axis (n is for the batch of the input samples, c is for the channel of the image of the input feature, h and w are for the height and the width directions of the feature image, respectively). $x_{nchw}$ is the feature image in pixel form before the normalization. |*I*| is the number of feature pixels in the feature image.

The general normalization is shown in Equation (2):

(2) $${\hat{x}}_{nchw}=\gamma \frac{x_{nchw}-\mu }{\sqrt{\sigma^{2}+\varepsilon }}+\beta$$

where *μ* and *σ^2^* represent the mean and variance of the sample data, respectively. *γ* is the scale of feature scaling in the normalization. *β* indicates the offset of the feature, defined as the average distance of the features. *ε* is a constant with a small value. As a stabilizing factor, *σ* prevents large fluctuations caused by small values. ${\hat{x}}_{nchw}$ is the normalized feature image.

#### 2.1.1. Batch Normalization

Batch normalization (BN) is a deep neural network training technique that makes the distribution of sample data in the batch consistent [2]. When training the neural network, BN divides the sample data into multiple small batches, then calculates the mean and variance of the feature map by a weighted average of these small batches to obtain the mean and variance of the entire sample data. The BN for fixed feature images with unchanged channels adjusts the mean and variance of the feature image in width and height directions and extracts the mean and variance of the feature image as shown in Equation (3):

(3) $$\mu_{\mathrm{BN}}=\frac{1}{NHW}\sum_{n,h,w}^{N,H,W}x_{nchw},\;\sigma_{\mathrm{BN}}^{2}=\frac{1}{NHW}\sum_{n,h,w}^{N,H,W}{\left(x_{nchw}-\mu_{\mathrm{BN}}\right)}^{2}$$

where N,H,W represent batch size, height, and width of the feature image, respectively.

Batch normalization requires that the mean and the variance of each batch are approximately the same with the entire sample data. It is suitable for scenarios where large batches and sample data distribution are relatively close.

#### 2.1.2. Instance Normalization

Instance normalization (IN) is often used to generate adversarial networks for image migration tasks [3]. IN for fixed feature channel and batch size adjusts the mean and variance of the feature image in width and height, as shown in Equation (4):

(4) $$\mu_{\mathrm{IN}}=\frac{1}{HW}\sum_{h,w}^{H,W}x_{nchw},\;\sigma_{\mathrm{IN}}^{2}=\frac{1}{HW}\sum_{h,w}^{H,W}{\left(x_{nchw}-\mu_{\mathrm{IN}}\right)}^{2}$$

It can be seen from Equation (4) that the IN method is equivalent to applying batch normalization to a single image and obtains the first-order statistics and the second-order statistics of the image. This method can handle image style transfer tasks with a relatively small sample size. As a method of training neural networks, it also has the advantages of fast convergence speed and real-time image generation. However, for different input features, for example, the characteristics of samples with different colors and sizes, IN may reduce the expressive ability of neural network when processing features. This method is also not suitable for tasks such as image classification, target detection, and instance segmentation with large data samples.

#### 2.1.3. Layer Normalization (LN)

LN is to normalize all neurons in an intermediate layer with fixed feature map batch dimension, to obtain the mean value and variance of the feature image through the channels, width, and height of the feature image. LN can calculate feature statistics through multiple channels, within a single piece of data without batch training required. LN extracts the mean and the variance of the feature image as shown in Equation (5) [4]:

(5) $$\mu_{\mathrm{LN}}=\frac{1}{CHW}\sum_{c,h,w}^{C,H,W}x_{nchw},\;\sigma_{\mathrm{LN}}^{2}=\frac{1}{CHW}\sum_{c,h,w}^{C,H,W}{\left(x_{nchw}-\mu_{\mathrm{LN}}\right)}^{2}$$

where C is the number of channels.

#### 2.1.4. Self-Adaptation Normalization (SN)

SN switches norm sets of the characteristics of three methods: BN, IN, and LN [5], according to the changes in the characteristics of the processed sample data, to realize the dynamic adaptation of its weight control parameters. First, calculate the feature mean and variance corresponding to the three single normalization methods [10]. Weight the obtained mean and variance through learnable weight parameters. The processing result is then regarded as the characteristic mean and variance of the self-adaptation normalization. The learnable weight parameters are determined by the control parameters obtained by the Softmax function, as shown in Equations (6)–(8) [11]:

(6) $$w_{k}=\frac{e^{\lambda_{k}}}{\sum_{k\in \left\{\mathrm{BN},\mathrm{IN},\mathrm{LN}\right\}}e^{\lambda_{k}}}$$

(7) $$\mu_{\mathrm{SN}}=w_{\mathrm{BN}}\times \mu_{\mathrm{BN}}+w_{\mathrm{IN}}\times \mu_{\mathrm{IN}}+w_{\mathrm{LN}}\times \mu_{\mathrm{LN}}$$

(8) $$\sigma^{2}{}_{\mathrm{SN}}=w_{\mathrm{BN}}\times \sigma^{2}{}_{\mathrm{BN}}+w_{\mathrm{IN}}\times \sigma^{2}{}_{\mathrm{IN}}+w_{\mathrm{LN}}\times \sigma^{2}{}_{\mathrm{LN}}$$

where *λ_k_* indicates the characteristics of the independent variable sample; $w_{k}$ are the weights; and k is used to indicate the selected normalization method. SN chooses different normalizers for different normalization layers of deep neural networks and learns their importance weights in an end-to-end way.

Integrating the advantages of common single normalization methods, SN has a stronger generalization ability to adapt to various network structures and tasks. In addition, SN has no sensitive hyperparameters, and, for different numbers of batches of sample data, it also shows good stability and accuracy.

## 3. Methodology

The shallow network of the CNN is responsible for extracting low-level texture information, such as color, lighting, and background of the image. The deep network extracts high-level semantic information, such as the target and layout of the image [12]. Absorbing the advantages of BN, SN has strong robustness against multi-features and the high-level texture features of the image. The IN is robust to low-level texture features. The SN normalization method uses the Softmax function to output learnable weights, and use the $\lambda_{k}$ in Equation (6) to combine the contributions from each normalization method to feature processing. Because it is composed of exponential form, the SN will assign weights of the normalization methods with a contribution of the mean equal to 0; therefore, the weight assignment of the normalization methods of each category will appear as an averaging effect. This will reduce the weight of the main normalization, leading to the secondary normalization method seriously affecting the feature information, which is supposed to be processed by the primary normalization method [13]. Regarding this issue, this paper proposes a weight learning method based on the adaptive weight loss layer, replacing the Softmax function in the original SN and making the weight value more reasonable [14].

Learning weights based on adaptive weight loss layer do not force the network to learn the three normalization methods equally; instead, it adaptively tunes the weights of the three normalized tasks according to the importance of them. This importance of a normalization method is estimated according to the generalization degree of the network model on the sample validation set. Network normalization weights [15] are updated through training samples. When the number of iterations is a multiple of a constant K, the weights are found by calculating the average loss of the current and previous K iterations, denoted as Pre_mean and Cur_mean in Equation (9), respectively. Then, subtract them to initially get the loss change rate between different normalization methods. This rate of change is considered as the trend of the verification loss in K iterations, as shown in Equation (9) [16,17,18,19]. The weight vector λ in Equation (12) is determined by the parameters Norm_trend and Norm_loss obtained in Equations (10)–(11):

(9) $$trend=\frac{Cur\_mean-Pre\_mean}{Cur\_mean}$$

(10) $$Norm\_trend=\frac{trend}{mean(trend)}$$

(11) $$Norm\_loss=\frac{Cur\_mean}{mean(Cur\_mean)}$$

(12) λ=Norm_trend×Norm_loss

During the training of the entire network, the weight vector *λ* is updated every K iteration. The updated weights are used to calculate the loss of training data and update the network parameter *θ* in the reverse channel [20], as shown in Equation (13):

(13) $$\theta =\arg\min\sum_{j=1}^{M}\sum_{i=1}^{N}\langle \lambda_{j},(\psi_{j}(I_{i};\theta )-L_{ij})\rangle$$

where *θ* is the optimized parameter set of the normalized prediction network. *L* is the loss function defined as the difference between the predicted value and the ground truth, using the squared error loss for comparison. *λ* and *θ* are initialized as vectors with all elements equal to 1.

The main advantage of this method is the adaptive learning of the weights of the three normalization methods through the weighted loss layer, which is summarized as follows: ResNet101 is used as the basic network framework, then an appropriate amount of images is chosen from each batch of samples for training and verifying loss calculations. The forward channel propagates the input vector forward through the network layers. After normalization, the output value is compared with the mathematical expected output, and the difference is considered as the loss function of the training error. The weight calculation module dynamically updates the weight of each normalization method.

**Figure 1:**
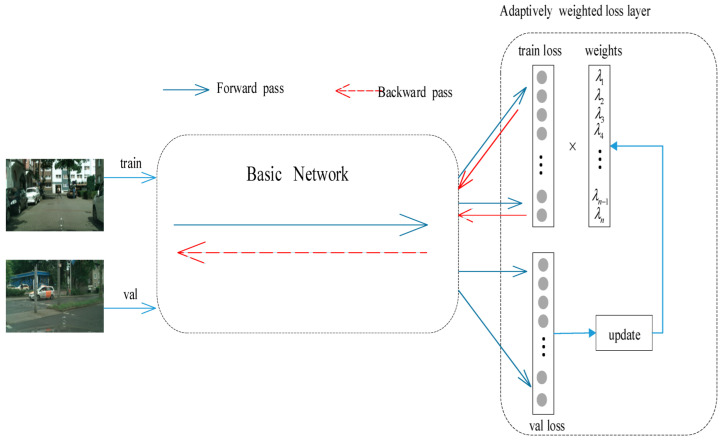
The structure diagram of the weight learning method of the adaptive weight loss layer.

Figure 1 shows the diagram of the proposed method. For faster convergence of the network training stage, to prevent the training loss curve from continuously oscillating in a small range, the features are normalized. In the model training phase, *λ_k_* is updated with the backpropagation of the training loss. To smooth the loss curve, according to the backpropagation of the loss function to the gradient of *λ_k_* and the change of the loss function, the *λ_k_* is updated along the direction of the gradient. The network will choose at least one primary normalization method to stabilize the loss during the training phase. The other two act as auxiliary normalization, under the condition of not affecting the main normalization method, by appropriate weights assigned to them. Because the normalization has no negative impact on features during the process of feature scaling, translation, and restoration, if a certain normalization method performs poorly on the robustness of the features extracted by the convolutional layer; then, its weight is reduced according to the loss function gradient. When dealing with shallow features, since IN has good robustness at this case, it is used as the primary normalization method, so the weight of IN is larger than BN and LN, and all weights are learned through backpropagation. When extracting deep features, the weight of BN is relatively large, which improves the expression ability of the proposed self-adaptive normalization after feature processing [21].

## 4. Experiments

Experiments are designed to demonstrate the multi-feature robustness of the proposed self-adaptive normalization, ensuring that the features extracted by each layer of the network can be properly processed [22]. Based on YOLAT [23], an instance segmentation model using traditional batch normalization, a weight loss layer is added and the proposed self-adapting mechanism is applied. Making use of the advantages of existing normalization methods (the small-batch robustness and shallow texture feature processing capabilities of IN, the advanced semantic feature processing capabilities of BN, and the generalization capabilities of LN), the proposed adaptive normalization further improves the task accuracy of the instance segmentation model and the convergence speed.

### 4.1. Model Training

In the experiments, the open-source deep learning framework Pytorch is used to implement the weighted loss layer and train the network. The ResNet101 + FPN network is used for basic feature extraction with end-to-end training. The COCO data set is divided into the training set, validation set, and test set according to YOLACT’s method. The model is optimized on the training set according to the performance, and the pros and cons of the model are analyzed and evaluated in the test set.

All the experiments are carried out in the Ubuntu 16.04 operating system. The server uses Intel Xeon Silver 4110 2.10GHz 8-core CPU, equipped with two Hynix 64 GB DDR4-2666 MHz memory, GTX2080TI 11G graphics card. The end-to-end model is used for all experiments. The interval K is set to 200. The stochastic gradient descent algorithm is used. Dropout uses a fully connected layer, and the ratio is set to 0.5. The basic learning rate is set to 0.0001, gradually decreasing by one-tenth after 70,000, 100,000, and 120,000 iterations.

### 4.2. Experimental Results and Analysis

In the experiments of this research, three network structures (ResNet-50, ResNet-101, and Darknet-53) are used as the convolutional network for feature extraction in YOLACT. The average accuracy in the COCO2014 verification set is recorded as average precision (AP) [24]. The experimental results comply with the commonly used evaluation standards for target segmentation in the COCO data-set. The AP is validated with intersection over union (IOU) threshold changing from 0.5 to 0.90 with a step of 0.05. For instance, the AP50 is the AP with IOU = 0.5. In this way, AP50~AP90 are the AP under nine IOU thresholds (0.5, 0.55, …, 0.90).

Besides the AP under various cross-combination conditions, we also count the AP for small targets (APS) with a size of less than 32 × 32 pixels, the AP of medium target (APM) of 32 × 32 and 96 × 96 pixels, and the AP for large targets (APL), which are larger than 96 × 96 pixels.

Each image in the training set was scaled to 400 × 400, 550 × 550, and 700 × 700 pixels according to the resolution, to compare the segmentation effects of different target image sizes.

**Figure 2:**
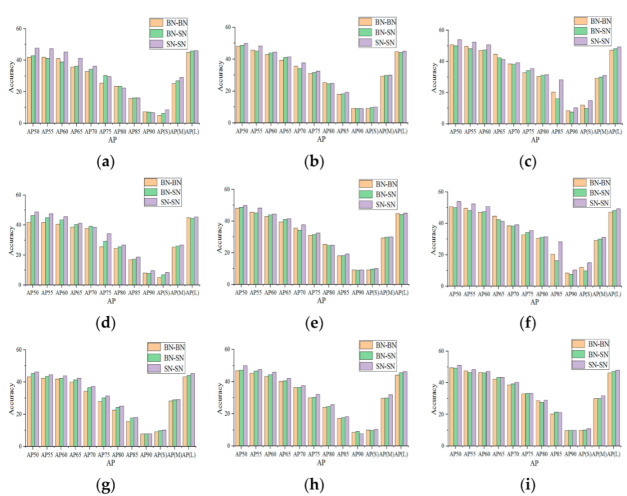
Average segmentation accuracy of three networks with different normalization methods.

The experimental results are shown in Figure 2, where the average test accuracies of instance segmentation with BN and SN normalization for three networks (ResNet-50, ResNet-101, and Darknet-53 [25,26,27,28]). The convolutional layer is divided into two parts: the first four convolutional layers are considered as the backbone, for feature extraction; the latter convolutional layer is the head for classification and position regression. Orange bars are the accuracy when BN is used in both the backbone and the head. Green bars represent the BN for the backbone but SN for the head. Purple means SN for both the backbone and the head.

Figure 2a–c represent the segmentation accuracy of the ResNet50 network, when the image sizes are 400 × 400, 550 × 550, and 700 × 700, respectively. The original algorithm YOLACT uses the BN normalization method in both the back-bone layer and the head layer. Comparing the combination of different normalization methods used in the backbone layer and head layer, it can be seen that using our proposed SN normalization method improves the accuracy of target segmentation to a certain extent. From Figure 2a–i, it can be seen that using our proposed SN normalization method in YOLCAT is better than using a single BN normalization method. When the backbone layers are ResNet50, ResNet101, and DarkNet53, the segmentation accuracy is improved. Figure 2d–f are the ResNet101 network with image sizes 400 × 400, 550 × 550, and 700 × 700. Figure 2g–i are the DarkNet53 network with image sizes of 400 × 400, 550 × 550, and 700 × 700. Comparing various normalization methods, it is found that using the proposed SN in the convolutional layer improves the accuracy of object segmentation to a certain extent. From Figure 2a–c, one finds that the SN improved the segmentation accuracy. It can be seen from Figure 2a,d that, as the number of network layers increases, the proposed SN has a good generalization ability. It can be seen from Figure 2b,e,h that, under different network structures, the proposed method also has good robustness to the accuracy of target segmentation. These experiments demonstrate that the proposed SN normalization method using adaptive weight learning has improved segmentation accuracy compared to the BN method alone (most green and purple bars are higher than the orange ones under the same setup). The normalization method we propose improves on the original normalization method in different networks and different image sizes. When both the backbone and head layers use SN, the segmentation accuracy of all large, medium, and small targets has been improved.

**Figure 3:**
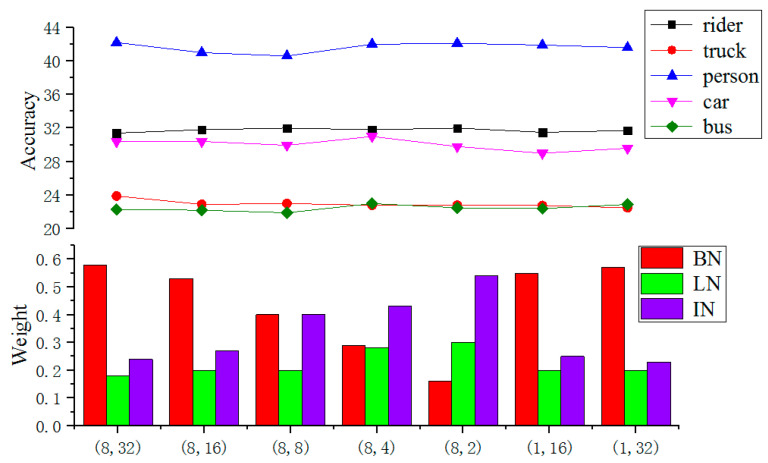
Improved segmentation accuracy regardless of the target under SN weight distribution.

Figure 3 compares average segmentation accuracy according to the weight coefficients of BN, IN, and LN, for five types of targets (rider, truck, person, car, and bus).

At the bottom of Figure 3, the first integer in the brackets represents the number of GPUs used, and the second one represents the number of samples per GPU, that is, batch size.

In Figure 3, the batch size is gradually decreasing from 32 until 2 in the first five experiments while keeping eight GPUs. From these experiments (the first five groups of the bar), one finds that the proposed adaptive weighting method is balancing the weights of different normalization methods according to the tasks and available resources. Specifically, the weight of BN is getting lower with the decrease of batch size; meanwhile, the weights of IN and LN are getting higher. On the contrary, with the increase of batch size for a single GPU setup (the last two groups of bars), when the resources are short, the weight of IN and LN is getting lower, and the weight of BN is getting higher. This confirms the idea of preferably weighting strong normalization methods according to their advantages under different batch size.

For various batch size, the segmentation accuracy of different types of targets is basically stable, as shown in the top portion of Figure 3. For instance, the segmentation of persons is about 42%. The rider and car targets are relatively close, stable at about 32%. The trucks and buses are around 22%. This means that, under the proposed SN method, regardless of the sample batch sizes, the segmentation has a stable accuracy. Under the same configuration, the improved self-adapting normalization performs very stably.

**Figure 4:**
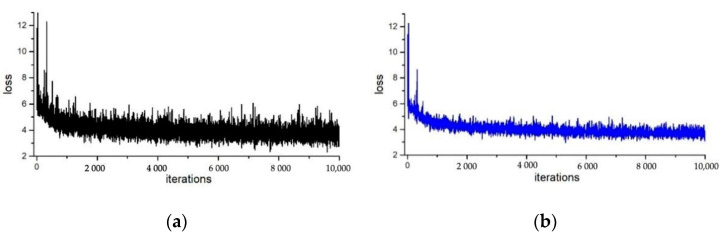
The loss curve of the ResNet50 network without and with the adaptive weighting.

**Figure 5:**
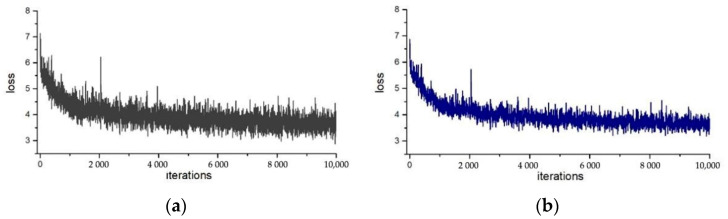
The loss curve of the ResNet101 network without and with the adaptive weighting.

Figure 4 and Figure 5 show the comparison of the loss curve in training of the segmentation mask, without and with the proposed normalization method applied in ResNet50 and ResNet101, respectively. The horizontal axis is the training iterations, while the vertical axis represents the loss function. Comparing the loss curves of (a) and (b) in Figure 4 and Figure 5, one finds that, after adding the adaptive weight loss layer to the network, the loss function values of the segmentation mask converge more steadily with smaller vibrations, especially in the later stage of the training. The overall loss value is also smaller. This means that the instance normalization method in SN can also speed up the convergence of the network.

Table 1 shows the comparison of the YOLACT algorithm combined with the pro-posed adaptive weight reduction layer and some related instance segmentation algo-rithms. The input image size of these experiments is all 550 × 550 pixels. The average segmentation accuracy of YOLACT using our proposed SN is 31.2%, which is 1.7% higher than that of BN-based YOLACT++ under the same conditions. The average precision of small target segmentation (APs) is increased by 0.1% (from 9.9% to 10%). The average precision of medium-sized targets (APm) is increased by 1.2%. The average precision of large targets (APl) is increased by 1.5%.

It needs to be noted that PA-Net, RetinaMask, FCIS, Mask R-CNN, and MS R-CNN algorithms obtain higher segmentation accuracy, but the frames per second (FPS) is much lower. In addition to the segmentation accuracy, the proposed method in this paper has higher efficiency leading to a higher FPS. Compared with the above algorithms, the segmentation accuracy of this method is slightly lower, but the FPS has been greatly improved, and it is more suitable for various real-time instance segmentation scenarios. Compared with the above algorithms, the segmentation accuracy is slightly lower for the proposed method, but it is more suitable for various real-time instance segmentation scenarios. In addition, it can be seen, from the above experimental comparison results, that the YOLACT algorithm based on the improved SN can guarantee the accuracy of segmentation at different stages. Comparing with the original algorithm (YOLACT++), the performance is improved. Our method can achieve real-time results with a loss of 0.8% accuracy, and can be used in scenarios with low accuracy requirements.

**Figure 6:**
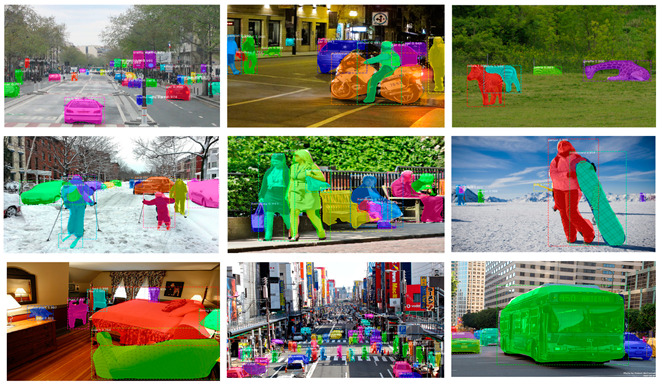
Visualization of YOLACT instance segmentation results based on an adaptive weight loss layer.

Figure 6 shows the performance of the proposed method in some typical practical scenarios. In day and night, indoor and outdoor, under various conditions such as general weather conditions, abnormal weather, and complex road conditions in the urban area, the segmentation obtained by this method is reliable. The targets in the complex background can be clearly and accurately identified. Considering the efficiency, the proposed method can be used in real-time applications.

## 5. Conclusions

This paper proposed a self-adaptive normalization method, in the form of an adaptive weight loss layer, which can be added to deep learning networks. The proposed method stabilized the training process of deep neural networks used in the instance segmentation tasks. The steadiness of the model training process is improved, and the training is accelerated. The proposed method solved two problems: (1) the problem of evenly distributed weights of the multiple normalization methods; and (2) the problem of feature processing contribution of 0 still assigned weights. The proposed adaptive weighting mechanism took the advantage of BN when the batch size is large, to make up for the shortcomings of other normalization methods. Especially in the visual task with limited batch size, the proposed method finds its advantages.

Different normalization methods used in object detection, instance segmentation, and video recognition rely on statistical information of different dimensions. The proposed SN method based on the adaptive weight loss layer tunes weights for different normalization layers of the neural network, which expands the boundaries of the normalization technology and is of great significance.

In addition, the adaptive normalization is robust under various batch conditions, which not only overcomes the batch normalization problem under sensitive conditions of small batches, but also makes the network training process steady under different batch sizes.

The high efficiency of the proposed method overperforms the state-of-the-art methods, which makes the real-time application possible. This method can also be extended to other different detection models and applied to different deep learning platforms.

## Figures and Tables

**Table 1:** Comparison of experimental results of different instance segmentation algorithms.

Method	Backbone	FPS	mAP	AP_50_	AP_75_	AP_s_	AP_m_	AP_l_
PA-Net	R-50-FPN	4.7	36.6	58.0	39.3	16.3	38.1	53.1
RetinaMask	R-101-FPN	6.0	34.7	55.4	36.9	14.3	36.7	50.5
FCIS	R-101-C5	6.6	29.5	51.5	30.2	8.0	31.0	49.7
Mask R-CNN	R-101-FPN	8.6	35.7	58.0	37.8	15.5	38.1	52.4
MS R-CNN	R-101-FPN	8.6	38.3	58.8	41.5	17.8	40.4	54.4
YOLACT++	R-101-FPN	33.5	29.5	47.0	30.6	9.9	30.1	46.2
YOLACT-SN	R-101-FPN	33.5	31.2	48.5	31.2	10.0	31.3	47.7
YOLACT-SN	R-50-FPN	45.0	28.2	46.6	29.2	9.2	29.3	44.8
YOLACT-SN	D-50-FPN	40.7	28.7	46.8	30.0	9.5	29.6	45.5
